# RNA-Seq analysis of gene expression changes triggered by *Xanthomonas oryzae* pv. *oryzae* in a susceptible rice genotype

**DOI:** 10.1186/s12284-019-0301-2

**Published:** 2019-06-24

**Authors:** Rezwan Tariq, Zhiyuan Ji, Chunlian Wang, Yongchao Tang, Lifang Zou, Hongda Sun, Gongyou Chen, Kaijun Zhao

**Affiliations:** 1 National Key Facility for Crop Gene Resources and Genetic Improvement (NFCRI), Institute of Crop Science, Chinese Academy of Agriculture Sciences (CAAS), Beijing, 100081 China; 2 School of Agriculture and Biology, Shanghai Jiao Tong University, 800 Dongchuan Road, Shanghai, 200240 People’s Republic of China

**Keywords:** Transcriptomics, *Xanthomonas oryzae*, Rice, Susceptibility, Differentially expressed genes, Pathways

## Abstract

**Background:**

*Xanthomonas oryzae* pv. *oryzae* (*Xoo*) is a destructive disease in most of the rice growing regions worldwide. *Xoo* injects the transcriptional activator-like (TAL) effector protein into the host cell to induce the susceptibility (*S*) gene(s) for spreading the disease. In the current study, a susceptible rice genotype, JG30, was inoculated with wild *Xoo* strain PXO99^A^ and its mutant PH without any TAL effector, to retrieve the differentially expressed genes (DEGs) having a role in susceptibility.

**Results:**

RNA-Seq data analysis showed that 1143 genes were significantly differentially expressed (*p*-value *≤*0.05) at 12, 24, 36 and 48 h post inoculation (hpi). Expression patterns, evaluated by quantitative real-time PCR (qRT-PCR), of randomly selected eight genes were similar to the RNA-Seq data. KEGG pathway classified the DEGs into photosynthesis and biosynthesis of phenylpropanoid pathway. Gene ontology (GO) analysis categorized the DEGs into the biological pathway, cellular component, and molecular function. We identified 43 differentially expressed transcription factors (TFs) belonging to different families. Also, clusters of the DEGs representing kinase and peroxidase responsive genes were retrieved. MapMan pathway analysis representing the expression pattern of genes expressed highly in biotic stress and metabolic pathways after PXO99^A^ infection relative to PH.

**Conclusions:**

DEGs were identified in susceptible rice genotype inoculated with PXO99^A^ relative to mutant strain PH. The identified 1143 DEGs were predicted to be included in the different biological processes, signaling mechanism and metabolic pathways. The Jasmonic acid (JA) responsive genes were identified to be downregulated in PXO99^A^ infected leaves. This study would be useful for the researchers to reveal the potential functions of genes involved in the rice susceptibility to PXO99^A^ infection.

**Electronic supplementary material:**

The online version of this article (10.1186/s12284-019-0301-2) contains supplementary material, which is available to authorized users.

## Introduction

Rice is a widely consumed crop, directly feeding more people than any other crop. It is the staple food in Asia, Africa and Latin America. Although rice in its various forms, has been under intense cultivation for more than 6000 years, it represents a laboratory for the investigation of bacterial diseases (Khush 1997). Currently, bacterial blight is a devastating disease in most of the rice growing regions, caused by the gram-negative bacterial pathogen, *Xanthomonas oryzae* pv. *oryzae* (*Xoo*) (Gnanamanickam et al. 1999). Naturally, *Xoo* enters inside the rice leaf through hydathodes of the leaf margin and multiplies into the intercellular spaces of epithelial tissues, then moves to the xylem vessel for systemic infection (Noda and Kaku 1999).

During infection, pathogenic *Xoo* injects transcriptional activator-like (TAL) effector protein via type III secretion system into host plant cell to promote the disease (Boch et al. 2009). The TALE may activate the susceptible (*S*) gene contributing to the disease progression or trigger the resistance (*R*) gene resulting to activate the host defense mechanism against pathogen. Interestingly, natural TAL effector proteins have conserved repeat region of 34 amino acids repeat, an N terminus region for type III secretion system, C-terminus containing acidic transcription activation domain and nuclear localization signal (Boch et al. 2009). Central conserved repeat region of TAL effectors determines the targeted genes in the host plant cell. The nucleotide specificity between TAL effector and host DNA sequence is determined by the number and order of 34 amino acids repeats (Moscou and Bogdanove 2009). Crystal structure elaborated that twelfth amino acid stabilizes the loop and thirteen amino acid residue interacts with the targeted nucleotide, projecting into the major groove (Boch and Bonas 2010; Deng et al. 2012).

Single immune receptor activates the different genes in host plant cell that need to be modulated. It was found that transcriptional reprogramming is a major feature of plant immunity and is governed by TFs and co-regulatory proteins associated within discrete transcriptional complexes (Moore et al. 2011). Upon immune receptor activation, selected TFs integrate with different signaling pathways in various ways. Among all TFs, *Os*WRKY exhibit significant role against various diseases from seedling to adult stage of the plant through induction of the salicylic acid (SA), jasmonic acid (JA), abscisic acid and gibberellic acid pathways (Jimmy and Babu 2015). For example, *OsWRKY45–2* exhibited *Xoo* resistance resulted in increased accumulation of JA (Tao et al. 2009); whereas, MYB TFs have promising importance in biotic resistance. Several MYB TFs have been reported with function in plant immunity, e.g., *AtMYB30*, *AtMYB44*, *AtMYB108* in Arabidopsis, and *HvMYB6* in barley (Ambawat et al. 2013; Chang et al. 2013). Moreover, boosting the innate immune response, plants produce different reactive oxygen species (ROS) to restrict the pathogen entry into the plant cell through the strengthening of the cell wall and cellulose deposition (Torres et al. 2006). Peroxisomes are the major sites for the accumulation of H_2_O_2_, as a result of the oxidative metabolism. For instance, NADPH oxidase and peroxidase class III are key proteins in ROS generation during the “oxidative burst” initiated in early defense reactions (Tripathy and Oelmüller 2012).

It is quite interesting to explore the genetic bases of the susceptibility in rice in response to the wild and mutant strains of *Xoo* at different time points. In the recent years, the rapid development in omics technology including transcriptomics has emerged a tremendous understanding of global gene networks to different diseases. RNA-Seq is considered unbiased technology, used to detect the DEGs with a broader dynamic range of expression level (Wang et al. 2009). The objective of the present study was to elucidate the DEGs at different time points in a susceptible rice genotype, JG30, after inoculation of the wild and mutant strains of *Xoo*, PXO99^A^ and PH strains, respectively. PXO99^A^ was isolated in the Philippines, and it is virulent toward a large number of rice varieties representing the diverse genetic resources of resistance. Unlike PXO99^A^, PH is a TALE free mutant of PXO99^A^ (Ji et al. 2016). Comprehensive analysis of RNA-Seq data identified several DEGs; moreover, there were different clusters of the DEGs, i.e., TFs, peroxidase responsive genes etc., were retrieved, involved in different biological pathways.

## Results

### Transcriptome profiling of JG30 genotype for DEGs

**Fig. 1:**
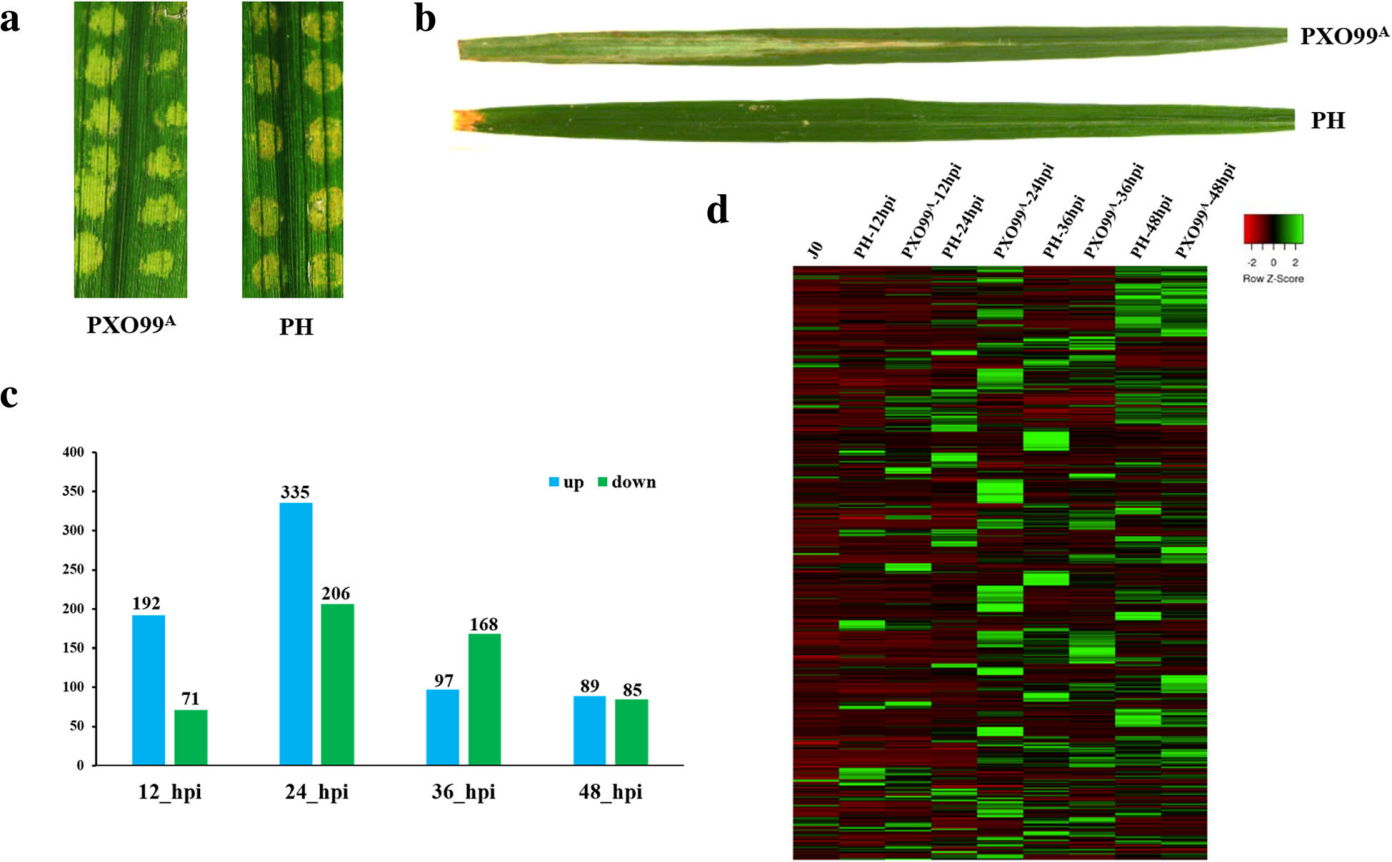
Comparison of the disease lesions and DEGs in JG30 genotype after PXO99^A^ and PH inoculations. **a** Disease symptoms in JG30 genotype after PXO99^A^ and PH inoculations via needleless syringe. Photographs were taken after 4 days of inoculation. **b** Inoculation of PXO99^A^ and PH strains into JG30 genotype by scissor dipped method. Photographs were taken 2 weeks of post inoculation. **c** Significantly expressed DEGs at different time points in PXO99^A^ inoculated leaves comparative to PH strain. The up and downregulated genes are shown in blue and green bars, respectively. **d** Heat map illustrating the expression pattern of DEGs at different time points after PXO99^A^ and PH infection

RNA isolated from the JG30 leaves inoculated with PXO99^A^ and PH by needless syringe, were subjected to RNA-Seq (Fig. 1a); additionally, healthy leaves of JG30 plants were infected by PXO99^A^ and PH strains via scissors dipped inoculation method for confirmation of the reaction pattern; JG30 is highly susceptible to PXO99^A^ but not to PH (Fig. 1b). Summary of the RNA-Seq analysis and depicted results are given in the Additional file 1: Table S1. Raw reads were ranged from 43,590,580 to 57,064,782. After the low-quality reads and adapter sequences were trimmed, the clean readswere ranged from 42,414,410 to 54,514,722. The clean reads were mapped to the available reference genome of rice using HISAT2 (2.1.0). Approximately, 79 to 84.42% reads were successfully mapped to the rice reference genome of Nipponbare. Given the high genome coverage of the Illumina sequencing reads, we depicted that the RNA-Seq data are useful for further deep bioinformatics analysis.

To determine the expression pattern of the JG30 genotype after PXO99^A^ and PH inoculations, a pairwise comparison between PXO99^A^ vs PH strains was made at a specific time point. A threshold level of *p*-value was set to 0.05 and revealed 1143 significant DEGs between PXO99^A^ vs PH at different time points (Additional file 1: Table S2). An overview of the comparative analysis between PXO99^A^ vs PH revealed that there were more up-regulated genes than downregulated genes (Fig. 1c, d). Briefly, there were 263 (192 upregulated and 71 downregulated), 541 (335 upregulated and 206 downregulated), 265 (97 upregulated and 168 downregulated), and 174 (89 upregulated and 85 downregulated) DEGs were identified at 12, 24, 36 and 48 hpi in PXO99^A^ vs PH.

### Identification of the differentially expressed TFs

**Fig. 2:**
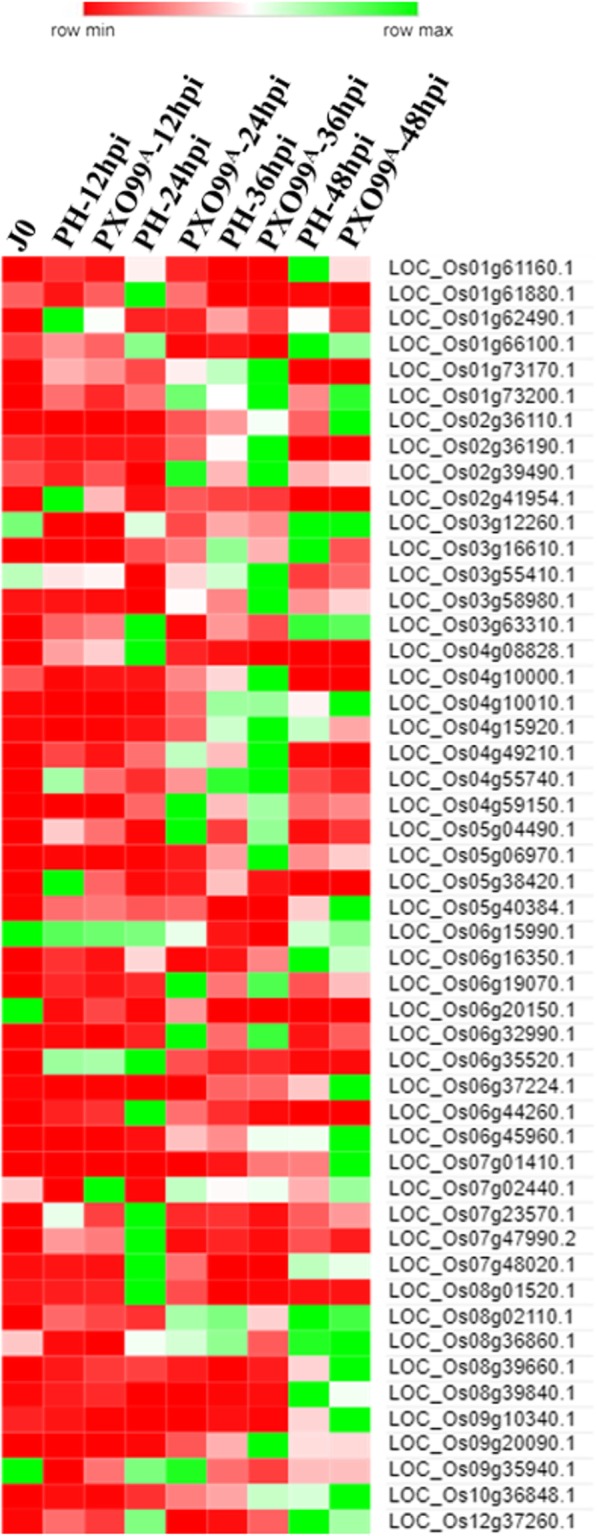
Heat map exhibiting the expression patterns of differentially expressed TFs at different time intervals after PXO99^A^ and PH infection in JG30 genotype. Gene IDs and name of TFs were retrieved from RGAP and cross-checked to plant TFDB

TFs are the key players, reported as differentially expressed in plants as a response to bacterial, fungal and viral infection (Amorim et al. 2017). In our experiment, 43 differentially expressed TFs, belonging to different TF families were retrieved (Fig. 2 and Additional file 1: Table S3). Among 10 differentially expressed TFs at 12 hpi, three TFs which belongs to the AP2-ERF (LOC_Os03g08490.1), Dof (LOC_Os06g17410.1) and mitochondrial-related TF (LOC_Os03g24590.1) were found to be upregulated in PXO99^A^ inoculated leaves of JG30 genotype than that of PH. At 24 hpi, six AP2-ERF, two bZIP, one bHLH, two NAC, two MYB, four WRKY, and two zinc finger domain related TFs were upregulated in PXO99^A^ vs PH. At 36 hpi, only two TFs belonging to the C2H2 (LOC_Os07g40300.1) and heat shock (LOC_Os10g07210.1) related TFs were upregulated; however, five TFs of different families were downregulated in PXO99^A^ inoculated leaves. Furthermore, four out of five differentially expressed TFs including one AP2-ERF (LOC_Os04g52090.1), two NAC (LOC_Os11g05614.1 and LOC_Os07g37920.1) and one C2H2 (LOC_Os03g55540.1) belonged to the downregulated genes in PXO99^A^ vs PH; whereas, only one TF of AP2-ERF (LOC_Os01g04800.1) was identified to be upregulated at 48 hpi. Shortly, among 43 TFs, 27 genes were upregulated and 16 genes were downregulated in PXO99^A^ inoculated leaves relative to the PH. Hence, upregulated genes may be involved in the susceptibility after PXO99^A^ infection.

### Identification of the kinase and peroxidase responsive genes in RNA-Seq data

**Fig. 3:**
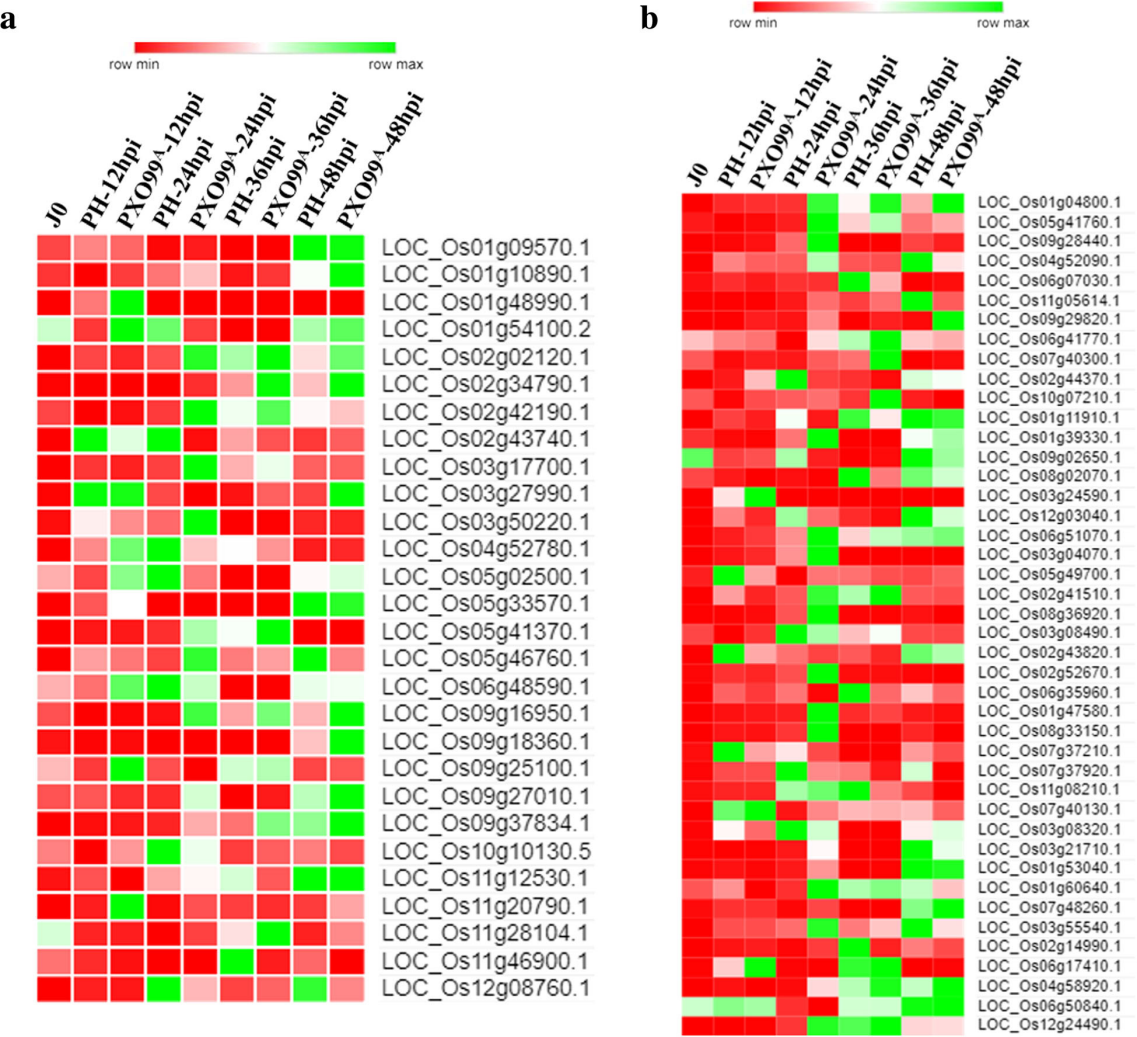
Heat maps demonstrating the expression patterns of the peroxidase and kinase responsive genes at different time points in JG30 genotype after PXO99^A^ and PH infection. **a** Heat map showing the expression patterns of different kinase responsive genes after PXO99^A^ and PH inoculations in JG30 genotype. The gene IDs were retrieved from the RGAP database. **b** Heat map of significantly expressed peroxidase responsive genes at different time points in JG30 genotype

Kinases form the largest gene family of the receptors in plants and have an important role in recognizing pathogen-associated molecular patterns and modulating the plant immunity in response to the invasive pathogen. We identified 28 significant differentially expressed kinase responsive genes at different time points (Fig. 3a and Additional file 1: Table S4). At 12 hpi, 10 DEGs (nine upregulated and one downregulated) were identified; among the nine upregulated DEGs, one adenylate kinase (LOC_Os11g20790.1), two MAPK (LOC_Os05g02500.1 and LOC_Os06g48590.1), one orthophosphate dikinase precursor (LOC_Os05g33570.1), two genes related to the protein kinase (LOC_Os01g48990.1 and LOC_Os04g52780.1), one casein kinase-like protein (LOC_Os01g54100.2), one CBL interacting protein kinase (LOC_Os09g25100.1), and one wall-associated kinase (LOC_Os10g10130.5) were retrieved to be upregulated in PXO99^A^ inoculated leaf samples relative to PH. In 24 hpi, 10 DEGs (nine upregulated and one downregulated) were identified. Among nine upregulated genes, including one pyrophosphate related kinase (LOC_Os01g09570.1), four protein kinase related genes (LOC_Os02g42190.1, LOC_Os03g50220.1, LOC_Os03g50220.1, LOC_Os09g16950.1, and LOC_Os09g27010.1), one MAPK (LOC_Os03g17700.1), one KI domain interacting kinase (LOC_Os05g41370.1), and two serine/threonine kinase related genes (LOC_Os05g46760.1 and LOC_Os02g02120.1) were upregulated in PXO99^A^ vs PH. Moreover, four genes were upregulated and two genes were downregulated (LOC_Os11g12530.1 and LOC_Os11g46900.1) at 36 hpi. At 48 hpi, four genes were identified to be upregulated, including three protein kinase related genes (LOC_Os03g27990.1, LOC_Os09g18360.1, and LOC_Os09g16950.1) and one serine/threonine kinase (LOC_Os01g10890.1); however, one gene from phosphoenolpyruvate kinase was downregulated in PXO99^A^ relative to the PH. The upregulated genes might have played role in basal defense against PXO99^A^, but the defense was not strong enough to cope with the pathogen attack.

The peroxidase responsive genes are important in plants controlling different processes such as development, growth, response to biotic and abiotic stress, and programmed cell death (Bailey-Serres and Mittler 2006). In our RNA-Seq experiment, 50 significant differentially expressed peroxidase responsive genes were identified at different time points (Fig. 3b and Additional file 1: Table S5). Among the 50 genes, 29 genes were upregulated and 21 genes were downregulated. At 12 hpi, two DEGs (LOC_Os07g02440.1 and LOC_Os09g35940.1) were observed to be upregulated, and five DEGs (LOC_Os01g73200.1, LOC_Os02g41954.1, LOC_Os04g49210.1, LOC_Os04g55740.1, and LOC_Os05g38420.1) were downregulated in PXO99^A^ inoculated JG30 leaves relative to PH. At 24 hpi, 19 DEGs were upregulated and 14 DEGs were downregulated in PXO99^A^ vs PH. At 36 hpi, nine DEGs were upregulated and only two genes (LOC_Os06g15990.1 and LOC_Os08g36860.1) were downregulated. Nevertheless, at 48 hpi, six genes (LOC_Os05g40384.1, LOC_Os06g37224.1, LOC_Os07g01410.1, LOC_Os08g39660.1, LOC_Os09g10340.1, and LOC_Os10g36848.1) were upregulated and three genes (LOC_Os01g62490.1, LOC_Os03g16610.1, and LOC_Os08g39840.1) were downregulated in PXO99^A^ inoculated leaves than that of PH. It is depicted that downregulated peroxidase responsive genes might have role in the resistance against PXO99^A^ in rice.

### Pathway enrichment analysis

**Fig. 4:**
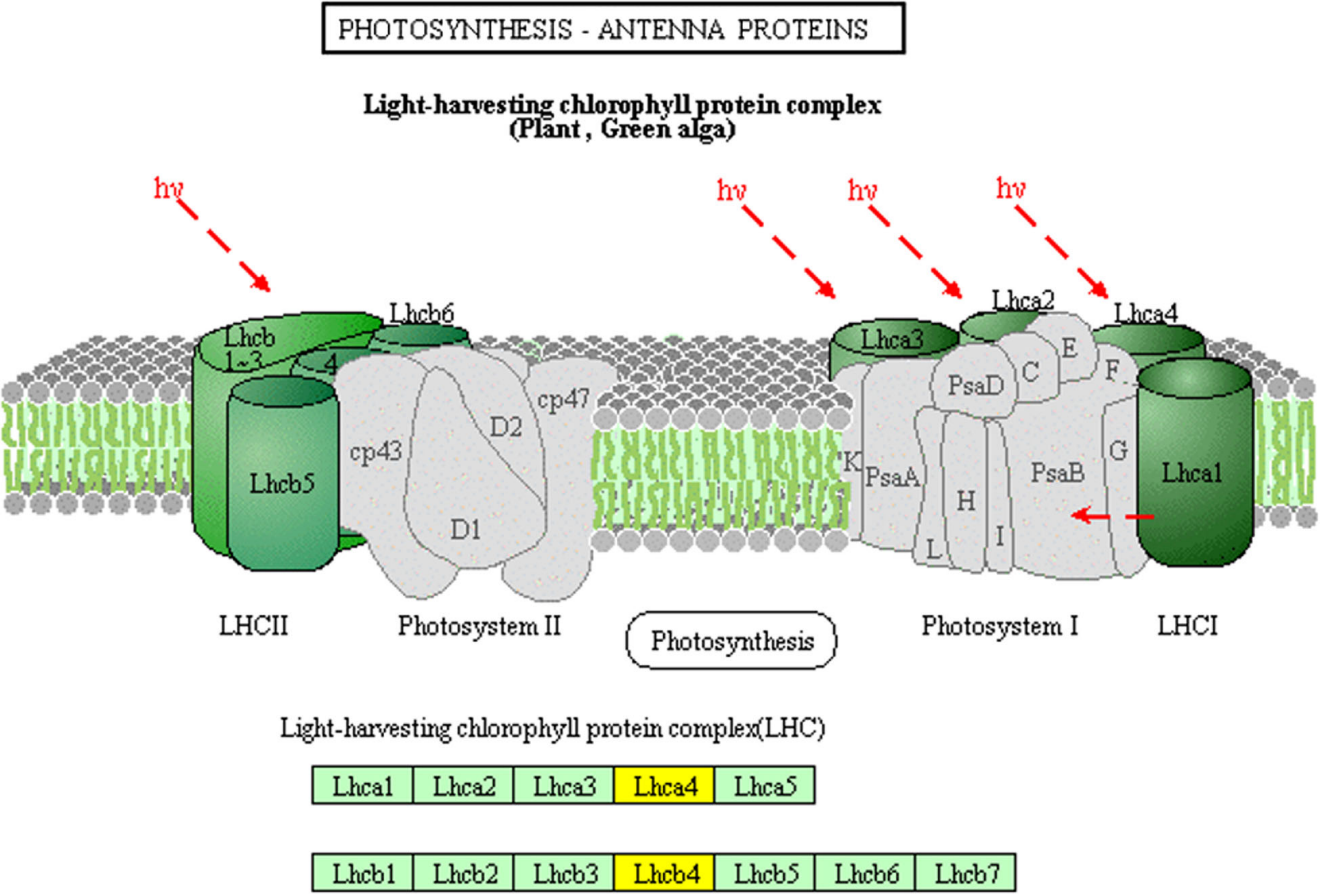
KEGG pathway analysis of the DEGs involved in photosynthesis pathway. Green boxes indicate the genes which express in rice, and the yellow color boxes show the DEGs downregulated in PXOO9^A^ relative to the PH inoculated leaves in JG30 genotype

We mapped the DEGs of JG30 genotype to the KEGG database to identify the significant pathways at different time points. The KEGG pathways were retrieved on the basis of *p*-value ≤0.05 (Additional file 1: Table S6). The “biosynthesis of phenylpropanoids” and “photosynthesis” were the prominent pathways at 12, 24 and 48 hpi. In photosynthesis pathway, the DEGs (LOC_Os07g37240.1, LOC_Os08g33820.1, and LOC_Os09g26810.1) were involved in the light harvesting chlorophyll (LHC) protein complex; Lhca4 and Lhcb4 were seemed to be downregulated in PXO99^A^ infected leaf samples of JG30 than that of PH (Fig. 4).

**Fig. 5:**
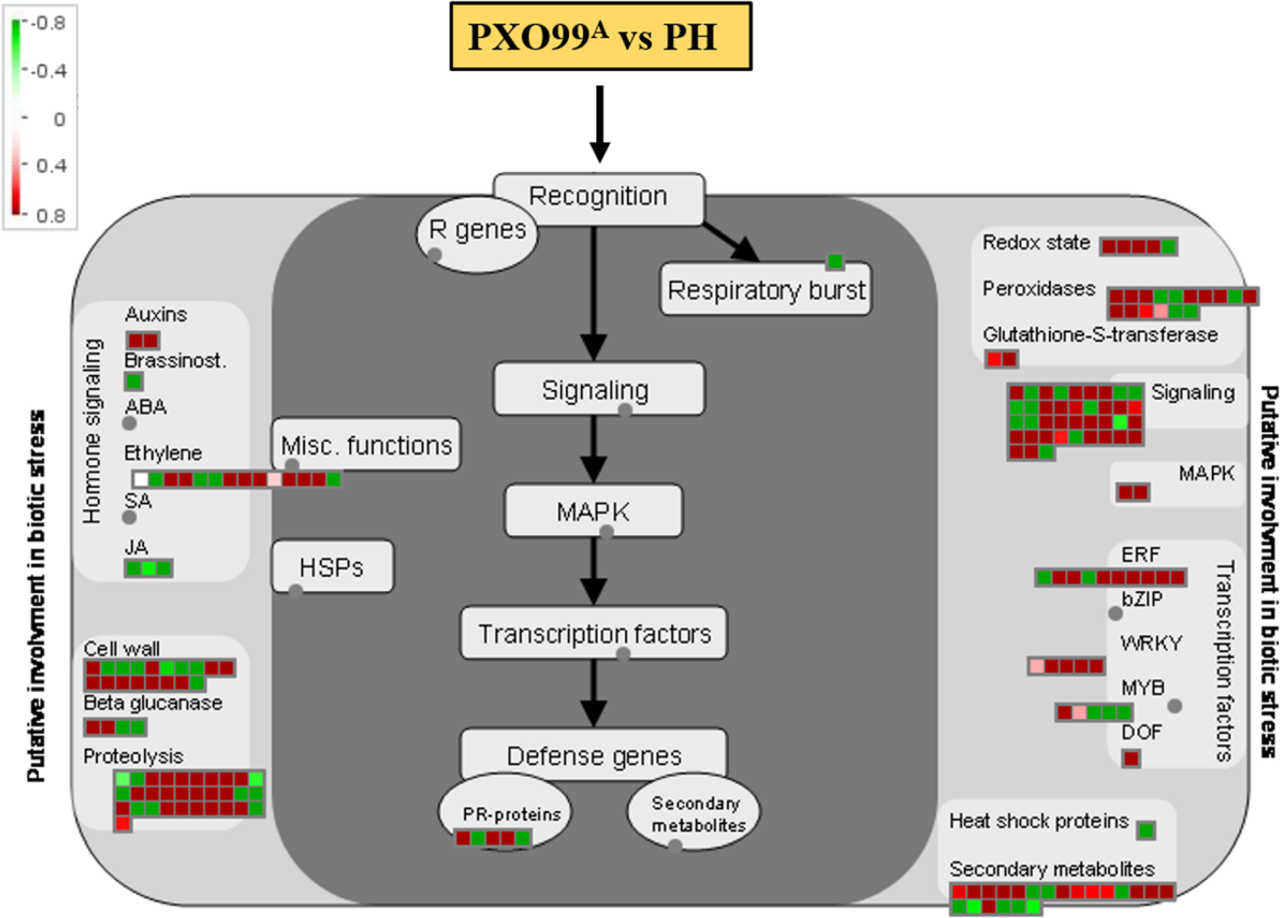
MapMan visualization of the DEGs involved in host-pathogen interaction. Each gene involved in biotic stress pathway is depicted by color signal where red signifies the genes expressed highly and green indicates the genes downregulated in PXO99^A^ vs PH. The intensity of the color is representing the level of expression

To elucidate the role of identified DEGs in biotic stress response, the MapMan package was employed to investigate the genes involved in plant-pathogen interactions. The input command of the DEGs was given in MapMan package to design a particular biological process using the rice annotation project database (RAP-DB). The DEGs with known functions, e.g., TFs, secondary metabolites, ethylene, proteolysis, and signaling are shown in Fig. 5. The detailed data are given in Additional file 1: Table S7. Briefly, most of the DEGs related to the peroxidase, redox state, signaling, and MAPK responsive genes were upregulated after PXO99^A^ infection relative to the PH strain. Eight out of ten ethylene responsive genes were identified as upregulated; moreover, all the five WRKY and one Dof responsive genes were upregulated in PXO99^A^ infected leaf samples, which indicates that these upregulated WRKY genes might have a key role in PXO99^A^ infection. The expression levels of 13 DEGs representing the secondary metabolites, three pathogenesis-related (PR) genes were influenced by PXO99^A^; these upregulated genes might have a role in the the susceptibility after PXO99^A^ infection in JG30 plants.

The two DEGs encoding auxins and nine ethylene-related genes were upregulated; on the contrary, five DEGs of ethylene, three JA and one brassinosteroids responsive genes were downregulated after PXO99^A^ infection. Afterward, 18 DEGs related to the cell wall were identified; among these 18 DEGs, 11 genes were upregulated and seven were downregulated in PXO99^A^ infected leaves as compared to the PH. Likewise, among the 31 proteolysis DEGs, 22 were upregulated and 9 nine genes were downregulated.

**Fig. 6:**
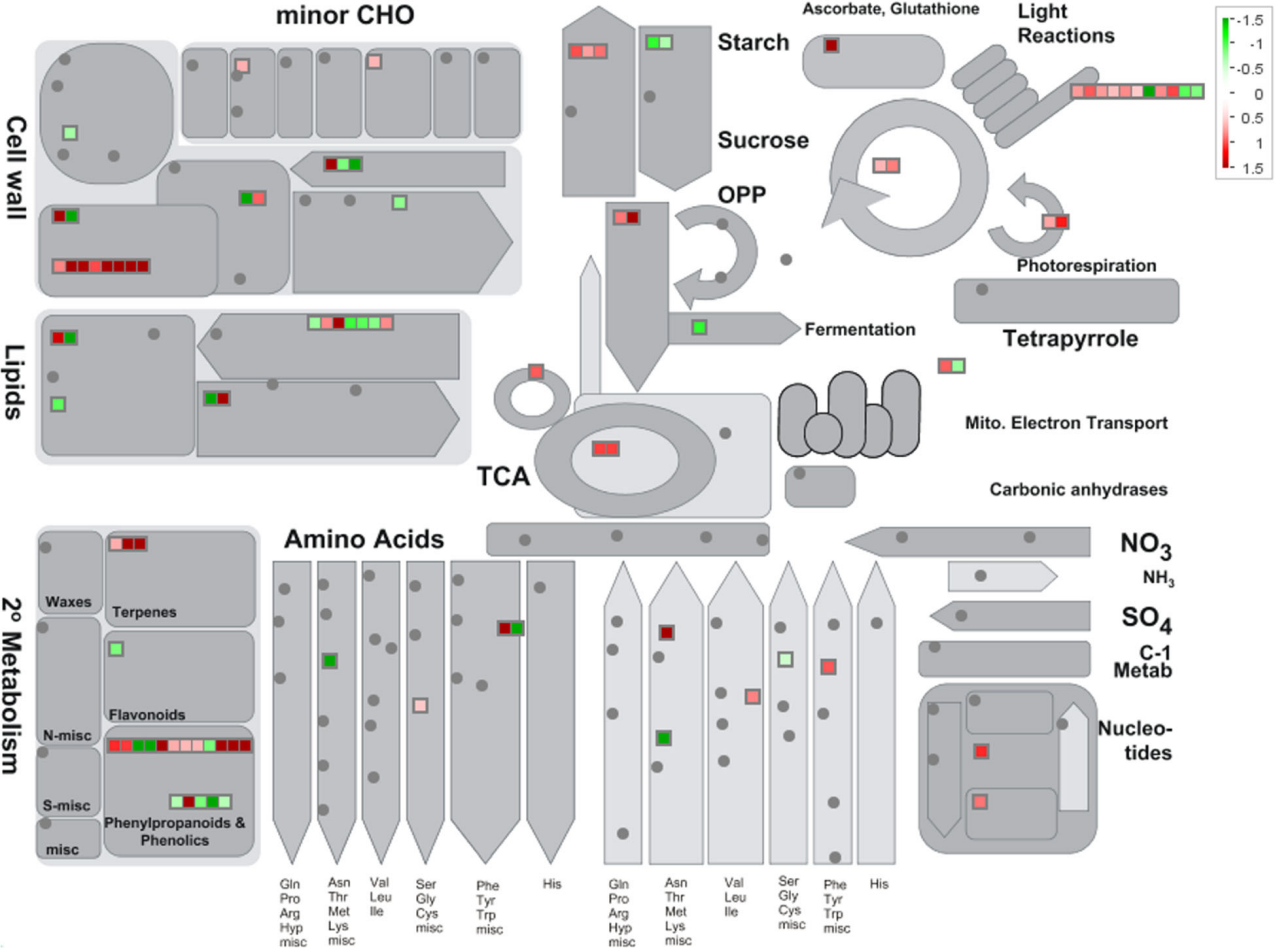
Overview of the metabolic changes in JG30 genotype after PXO99^A^ infection relative to PH, visualized by MapMan. DEGs with Log2FC *≥*1 or *≤ −* 1 were imported into MapMan tool. Up and down-regulated genes are represented in red and green squares, respectively

As an additional analysis to get a clear understanding of the participation of metabolic pathway in PXO99^A^ infection relative to the PH, MapMan package was used to classify the DEGs into metabolic pathways and processes (Fig. 6 and Additional file 1: Table S8). The genes having higher expression level are involved in the “light reaction” and “photorespiration” bins representing the photosynthesis category. Additionally, some genes encoding the “lipids”, “sucrose” and “starch” were upregulated in metabolic pathway. In secondary metabolism, most of the DEGs representing the “terpenes”, “phenylpropanoids and phenolics”, and “nucleotide metabolism” (ribonucleoside-diphosphate reductase) were upregulated in susceptibility conditions after PXO99^A^ infection. The visual annotations of the DEGs provided a valuable resource for the exploration of the pathways involved in susceptibility after PXO99^A^ infection.

### Gene ontology enrichment analysis

The GO analysis functionally characterizes the DEGs into three different categories, i.e., biological process, cellular component, and molecular function. The GO analysis was done using the AgriGO online tool. The GO analysis of all the DEGs is shown in the Additional file 1: Table S9. The significant GO terms after infection of PXO99^A^ and PH at each time point were retrieved using the false discovery rate (FDR) ≤ 0.05 (Additional file 1: Table S10). In PXO99^A^ vs PH at 12 hpi, the GO terms were significantly enriched in biological process (10), cellular component (6) and molecular function (7). At 24 hpi, 56 significant enriched GO were identified, including 28 biological processes, five cellular function, and 23 molecular function related terms. In PXO99^A^ vs PH at 36 hpi, the significant GO terms were classified into the biological process (5), cellular component (1) and molecular function (13). Unlike 36 hpi, only five enriched GO terms were identified, including biological process (4) and molecular function (1).

**Fig. 7:**
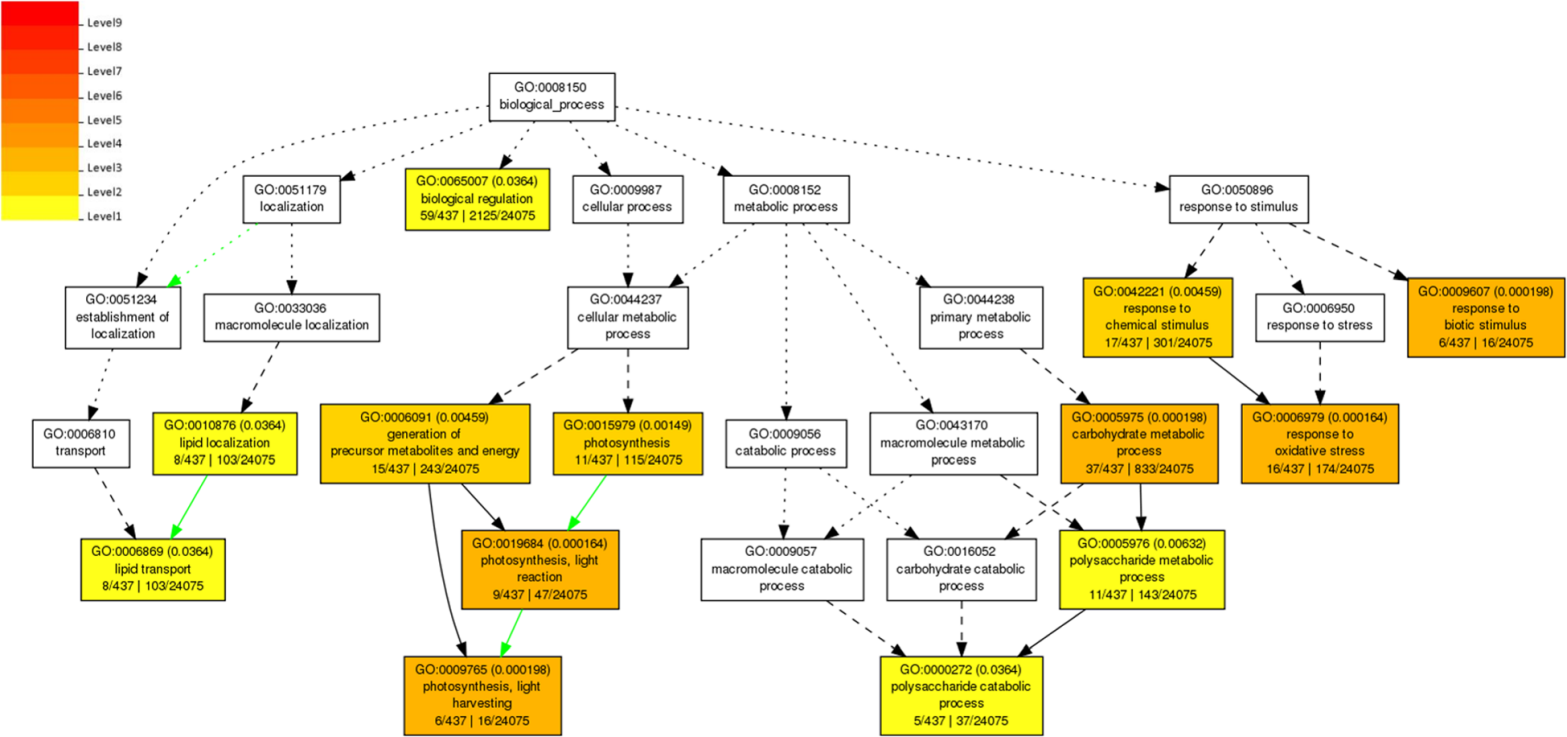
GO term of biological process exhibiting the clustering of DEGs with different coloring patterns involved in different biological processes. A key of different colors is mentioned exhibiting the significance level of the each GO term in the biological process

The significant biological process related GO term in all time points (12, 24, 36, 48 hpi) are mentioned as follows: “biological regulation (GO:0065007)”, “response to chemical stimulus (GO:0042221)”, “response to biotic stimulus (GO:0009607)”, “lipid localization (GO:0010876)”, “generation of precursor metabolites and energy (GO:0006091)”, “photosynthesis (GO:0015979)”, “carbohydrate metabolic process (GO:0005975)”, “response to oxidative stress (GO:0006979)”, “lipid transport (GO:0006869)”, “photosynthesis, light reaction (GO:0019684)”, “polysaccharide metabolic process (GO:0005976)”, “photosynthesis, light harvesting (GO:0009765)”, and “Polysaccharide catabolic process (GO:0000272)” (Fig. 7).

### RNA-Seq data validation

**Fig. 8:**
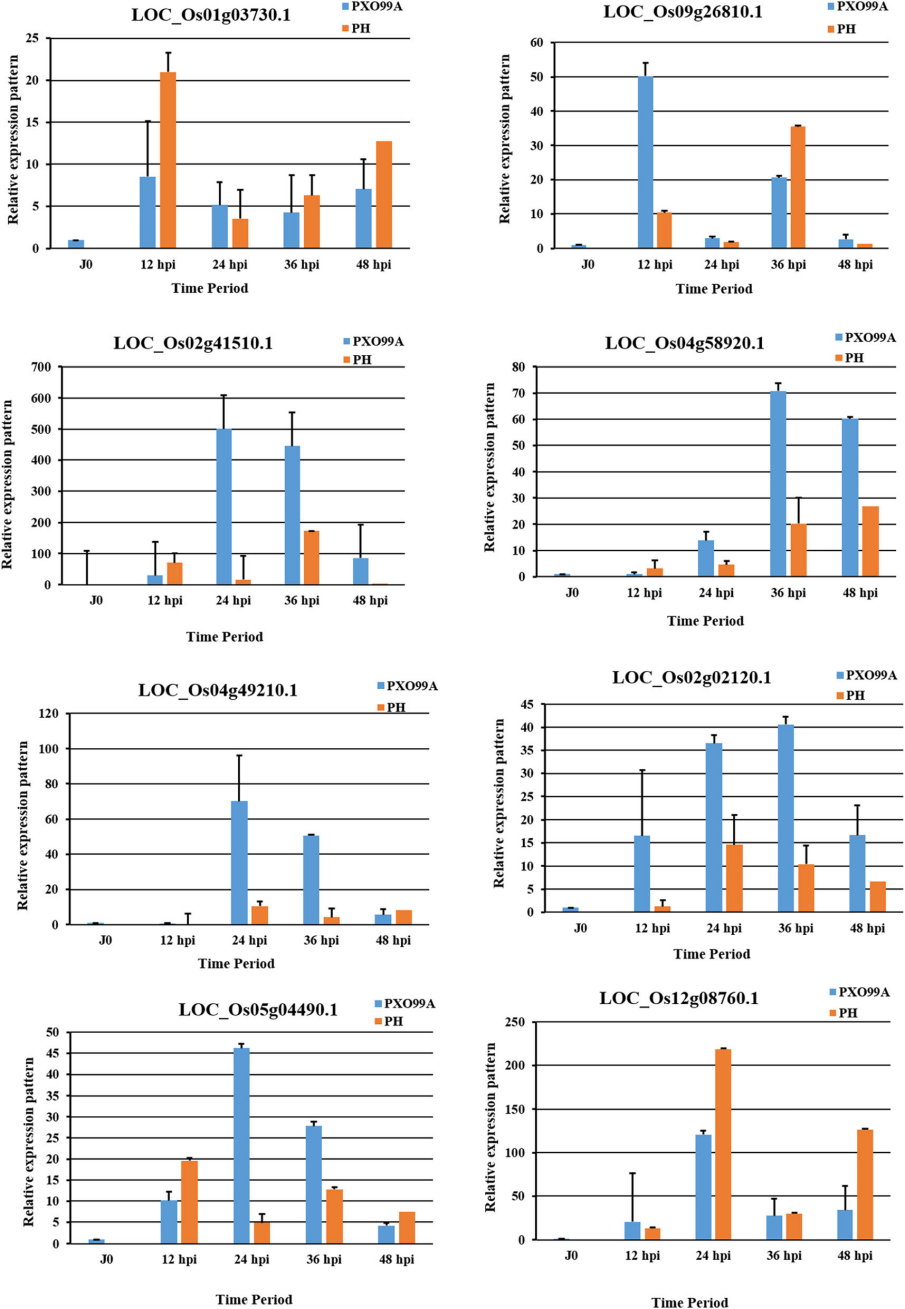
qRT-PCR based expression pattern of the eight randomly selected DEGs from RNA-Seq data. Ubiquitin was used as an internal control in reaction; data are represented as mean ± SD of three biological replicates

Eight randomly selected DEGs were evaluated for their expression patterns at different time points to validate the RNA-Seq data (Fig. 8). The gene sequences were retrieved from phytozome v12.1. LOC_Os01g03730.1, encoding nuclease I gene was highly expressed at 12 hpi in PH inoculated leaves of JG30 than that of PXO99^A^. LOC_Os09g26810.1, representing the type II chlorophyll binding protein was upregulated at 12 hpi in PXO99^A^ vs PH. Moreover, R2R3-MYB (LOC_Os02g41510.1) TF was exhibited to be overexpressed at 24 and 36 hpi in PXO999^A^ vs PH. LOC_Os04g58920.1 encoding the zinc finger domain-containing protein was induced at 24, 36 and 48 hpi in PXO99^A^ inoculated leaves relative to the PH. 2OG-Fe (II) oxygenase responsive gene (LOC_Os04g49210.1) was highly expressed at 24 and 36 hpi in PXO99^A^ vs PH, respectively. LOC_Os02g02120.1 and LOC_Os05g04490.1 representing the Serine/threonine kinase and peroxidase responsive genes, respectively, were upregulated at 24 and 36 hpi in PXO99^A^ vs PH. Pyruvate/Phosphoenolpyruvate kinase gene (LOC_Os12g08760.1) was overexpressed in PH infected samples as compared to the PXO99^A^. In short, the qRT-PCR results validated the expression pattern of selected DEGs mentioned in RNA-Seq data.

## Discussion

Plants are attacked by different pathogens that seriously damage their productivity. Plants response to these stresses by adopting different physiological, cellular and molecular mechanisms. A crucial step in plant defense against a pathogon is to timely counter the stress in a rapid and an efficient manner. In our RNA-Seq experiment, the JG30 leaves exhibited two distinct morphological symptoms after PXO99^A^ and PH infection. PXO99^A^ infection overcomes the PAMP-triggered immunity (PTI) owing to the fact that the secreted TAL effectors successfully interfere with or inhibit the defense response. However, the PH strain, without the TAL effector proteins, was countered by plant PTI and halted the infection. In detail, protein kinases are involved in mediating the different signaling process in the plant-pathogen interaction. The serine/threonine responsive genes (LOC_Os05g46760.1 and LOC_Os02g02120.1) were observed to be upregulated at 24 hpi in PXO99^A^ vs PH. The previous studies elaborated that serine/threonine kinase responsive genes are important in resistance against *Pseudomonas syringae* in tomato (Lin and Martin 2007). The most of the kinase responsive DEGs were upregulated in our experiment in susceptible leaves of JG30 after PXO99^A^ infection comparative to PH strain; on the contrary, kinase and signaling responsive genes are playing role in PTI as pattern recognition receptors (PPRs) and activate the plant defense system in a short time interval (<10 min) (Abramovitch et al. 2006; Pfeilmeier 2017); moreover, PRRs activation triggers the signaling responsive genes which further upregulate the expression of > 300 plant genes (Cheng et al. 2017; Thilmony et al. 2006). According to the literature, to counter the PTI, pathogens deliver the effector protein into the plant cell to suppress the basal defense. In our study, PXO99^A^ injected the TAL effector proteins through type III secretion system into the plant cell to suppress the PTI and induce ETS. In the absence of ETI, JG30 leaves showed susceptibility symptoms. Unlike PXO99^A^ inoculated samples, PH infected leaves exhibited resistance symptoms owing to the absence of effector protein in the PH strain. In a different transcriptome study, a rice genotype with *Xa23* gene, exhibited the resistance against PXO99^A^ in form of hypersensitivity cell death reaction (Tariq et al. 2018).

Likewise, plant immune receptors activate several genes to tackle the biotic stress and coordinate their stress response with growth to maximize their fitness. TFs interact with the *cis*- regulatory elements in the promoter regions of stress-related genes and up-regulate the expression of many genes to activate the biotic stress tolerance (Agarwal et al. 2010). In our experiment, *OsWRKY14* (LOC_Os01g53040.1), *OsWRKY17* (LOC_Os03g21710.1), and *OsWRKY21* (LOC_Os01g60640.1) were upregulated in PXO99^A^ infected leaves than that of PH. WRKY17 was exhibited to be a negative regulator of basal resistance to *Pseudomonas syringae* pv. *tomato* in *Arabidopsis thaliana* (Journot-Catalino et al. 2006). Whereas, overexpression of *OsWRKY21* was found to be involved in the expression of calcium and calcium/calmodulin genes, important in plants as a messenger in modulating diverse physiological processes for stress adaptation (Park et al. 2005). In wheat, WRKY14 was reported to be upregulated in resistance cultivar against yellow dwarf virus infection (Wang et al. 2013).

AP2-ERF TFs are unique to plants, playing a vital role throughout the plant life cycle, e.g., leaf epidermal cell identity and response to various biotic and environmental stresses (Riechmann and Meyerowitz 1998). AP2-ERF TFs activate the defense-related genes, i.e., *PR*, osmotin, beta-1,3-glucanase and chitinase responsive genes, in *Arabidopsis thaliana* under different biotic stress conditions (Moffat et al. 2012; Zarei et al. 2011); similarly, ERF proteins regulate the ethylene biosynthesis pathway in *Arabidopsis thaliana* against *Botrytis cinerea* (Zhao et al. 2012). Moreover, *OsERF922* was reported to negatively regulate the resistance to *Magnoparthe oryzae* in rice (Liu et al. 2012). AP2-ERF TFs might have regulated the *PR* response genes in our experiment.

The different MYB responsive TFs, i.e., MYB51 (LOC_Os08g33150.1) and R2R3-MYB (LOC_Os07g37210.1) were observed to be upregulated in the PXO99^A^ infected leaves of JG30. It was found that AtMYB51 is associated with wound response against insect herbivores (Johnson and Dowd 2004); The BOTRYTIS SUSCEPTIBLE1 gene encodes the MYB51, interacting with the signaling pathway in wound response, which ultimately triggers the peroxidase responsive genes (Mengiste et al. 2003). In another study, AtMYB51 was observed to regulate the indolic glucosinolate biosynthesis in leaves after pathogen attack (Gigolashvili et al. 2007). R2R3-MYB TFs control the wide variety of processes, including phenylpropanoid mechanism and secondary cell wall formation (Soler et al. 2015); in our study, R2R3-MYB TF (LOC_Os07g37210.1) may be negatively regulating the resistance mechanism against PXO99^A^ infection.

The positive upregulations of the two different NAC TFs, i.e., OsNAC95 (LOC_Os06g51070.1) and OsNAC22 (LOC_Os03g04070.1) were observed in PXO99^A^ infected leaves. In previous studies, *OsNAC22* was observed to be overexpressed in rice infected with rice dwarf virus and rice black-streaked dwarf virus (Nuruzzaman et al. 2015). We predict that OsNAC22 might be related to the response induced by PXO99^A^.

Likewise, the cluster of DEGs enriched in KEGG pathway related to the photosynthesis exhibited to be downregulated after PXO99^A^ infection than that of PH. Besides the KEGG pathway, the DEGs involved in the light reaction were enriched in MapMan metabolic pathway. Previous studies indicate that downregulation of the photosynthesis responsive genes reflects the usage of energy and resources to defend the invading pathogens (Jain et al. 2017; Yu et al. 2014). Pathogen infection restricts the photosynthesis activity and availability of nutrient sources for the pathogen (Berger et al. 2007); in *Arabidopsis*, the photosynthesis was decreased after *P. syringae* infection (Bonfig et al. 2006). Moreover, the genes involved in the photosynthesis reaction were repressed by *Xoo* in rice (Narsai et al. 2013). It was found in another study that *Rhizoctonia solani* infection leads to repressing the photosynthesis, increases the secondary metabolism, ROS accumulation and cell death in rice (Helliwell et al. 2013).

Plant hormones, JA and ET, play a diverse role in resistance against pathogens to a remarkable spectrum. In our experiment, the DEGs (LOC_Os12g37260.1, LOC_Os08g39840.1, and LOC_Os06g11290.1) representing the JA hormones were observed to be downregulated in PXO99^A^ inoculated leaves. It was observed that JA regulates the expression of *PR* genes in rice exhibiting the role in resistance to *M. oryzae* (Agrawal et al. 2000); furthermore, an increased accumulation of JA in rice was observed, causing resistance to *Xoo* (Tao et al. 2009). The upregulation of the ET responsive gene, *OsACS2*, encoding the allene oxide synthase illustrated the broad spectrum resistance to *M. oryzae* and *R. solani* (Helliwell et al. 2013).

The plant cell wall is the physical barrier to restrict the entry of pathogen, acting as a passive defense barrier. In our experiment, the cell wall-related genes were differentially expressed at different time points in PXO99^A^ vs PH. Downregulation and upregulation of the cell wall-related genes depicted to have an impact on abiotic and biotic stresses (Bacete et al. 2018). The cell wall component, lignin, was induced by different plant hormones that regulate the plant defense. In *Arabidopsis*, infection of *P. syringae* and *X.compestris* resulted in overexpression of lignin biosynthesis genes (Mohr and Cahill 2007). Similarly, hemicellulose is cell wall polysaccharides, such as xylans and xyloglucans that exhibited the resistance to *Plectosphaerella cucumerina* in *Arabidopsis* (Delgado-Cerezo et al. 2012).

Overall, the RNA-Seq data of different time points identified the DEGs. This study highlighted the genes differentially expressed only in susceptibility condition in rice after PXO99^A^ infection. The expression patterns of the genes expressed in susceptibility provide new information for researchers to explore the susceptibility mechanism conferred by PXO99^A^.

## Materials and methods

### Plant materials and growth conditions

The seeds of a rice genotype, JG30, were surface sterilized in 70% ethanol for 5 min and washed with sterilized water. Afterward, water soaked the sterilized rice seeds for overnight. After pre-germination, rice seeds were sown in pots and kept them in the green house of Chinese Academy of Agricultural Sciences, Beijing, and P.R. China. The condition of the green house in which rice seeds were grown was: 25/30 °C under a 14 h light /10 h dark cycle with 80% RH.

### Inoculation of different *Xoo* strains

Two different *Xoo* strains, PXO99^A^ and mutant of PXO99^A^ (PH), were used for inoculation. Particularly, PH strain is without the TAL effector gene, responsible for pathogenicity symptoms in the host plant. Initially, PXO99^A^ and PH were subcultured on TSA plate (tryptophan, 10 g/L; sucrose, 10 g/L; glutamic acid, 1 g/L and agar, 5 g/200 ml) for 48 h. The inoculum was prepared by suspending the bacterial strains in sterilized water and concentration was measured by determining the OD_600_ (Optical density at 600 nm) between 0.9 and 1.0. The leaves of JG30 genotype were infected by using scissors dipped in bacterial suspensions to clip leaves 1-2 cm down from the tip of the leaf blade. After 2 weeks of post inoculation, lesions were observed from the cut surface to the distal-most position of the leaf blade exhibiting water-soaked lesions. For RNA-Seq, both strains, PXO99^A^ and PH, were inoculated into 50 days old leaves of JG30 genotype by needless syringe. Inoculated leaves with three biological replicates were harvested at 12 hpi, 24 hpi, 36 hpi, and 48 hpi, respectively. The harvested leaves were immediately frozen in liquid nitrogen and stored at − 80 °C until RNA extraction.

### RNA extraction and Illumina sequencing

Total RNA of inoculated (12 hpi, 24 hpi, 36 hpi, and 48 hpi) and mock (J0) leaf samples were extracted through TRIZOL® reagent (TIANGEN, Beijing, China) according to the manufacturer^’^s protocol. Extracted RNA from different samples was purified by using RNase-free DNase I (TaKaRa, Kyoto, Japan) to remove the genomic DNA contamination. Total RNA concentration in different samples was calculated using NanoDrop microvolume spectrophotometer (Thermo Scientific NanoDrop Products, Waltham, MA, USA). Thereafter, the Illumina HiSeq2500 platform was used for RNA-Seq. cDNA library construction and sequencing were done by Novogene Bioinformatics Technology Co., Ltd., Beijing, China.

### Analysis of the RNA-Seq data

Quality reads of the raw RNA-Seq data were processed by the fastQC application v0.11.2 (Anders and Huber 2010); each paired-end data had the insert size 200–300 bp. The low quality reads and reads containing adapters were removed by the Trimmomatic (0.36.5) tool to get the clean reads data (Bolger et al. 2014). Afterward, paired-end clean reads were aligned to the available reference genome of rice (http://rice.plantbiology.msu.edu/pub/data/Eukaryotic_Projects/o_sativa/annotation_dbs/pseudomolecules/) using HISAT2 (2.1.0) (Kim et al. 2015). StringTie (1.3.4) was employed to count the number of reads mapped to each gene and quantification of the gene expression level in FPKM (number of fragments per kilobase of the transcript sequence per million base pairs sequenced) (Pertea et al. 2015). The differential expression analysis between PXO99^A^ and PH inoculated samples was performed using the DESeq2 R package (2.11.38). Genes with Log2 fold change (Log2FC) ≥1 (up-regulated) or Log2FC ≤1 (down-regulated) were considered as DEGs in comparative analysis.

### Functional classification and pathway enrichment analysis

The functional enrichment analysis including GO analysis was performed to identify which DEGs were significantly involved in each GO term. GO enrichment analysis was performed by AgriGO software (Du et al. 2010); GO term with FDR *≤* 0.05 was considered significantly enriched by DEGs. The KEGG pathway analysis was executed to retrieve the enriched pathways with *p*-value *≤*0.05. Additionally, MapMan package was employed to get the graphical representation of the DEGs playing role in biotic stress response and metabolic pathways (Thimm et al. 2004).

### Validation of RNA-Seq data

The expression pattern of the differentially expressed genes was done by qRT-PCR to validate the RNA-Seq data. The transcript sequences of the eight nominated genes were retrieved from Phytozome v12.1. The primers of the nominated genes were designed using AmplifX 1.5.4 software, and the primers used in the qRT-PCR were given in Additional file 1: Table S11. Ubiquitin was used as an internal control in qRT-PCR; the reaction was performed in a 96-wells plate on an ABI prism 7500 Real-Time PCR system (Applied Biosystem, Foster City, CA, USA) using SYBR Green Master ROX (TaKaRa). The relative expression level of the selected DEGs was calculated with the 2^-*ΔΔCT*^ method (Livak and Schmittgen 2001). The reaction was carried out using three biological replicates with three technical replicates.

## Conclusion

In this study, two different strains of *Xoo*, PXO99^A^ and PH, were inoculated into the JG30 genotype leaves to uncover the differentially expressed genes at different time points. A total of 1143 genes were differentially expressed in JG30 genotype. GO and pathway analysis revealed that DEGs were involved in biological regulation, response to biotic stimulus, response to oxidative stress, lipid transport, photosynthesis, and light reaction. The antenna responsive genes having role in the photosynthesis were downregulated in PXO99^A^ infected leaf samples. In plant-pathogen interaction pathway, JA, brassinosteroids and ethylene responsive genes were downregulated in PXO99^A^ infected leaf samples than that of the PH. Moreover, most of the genes representing the cell wall and secondary metabolites were downregulated in susceptibility condition. We also identified the genes of different TF families, kinases and peroxidase responsive genes that were differentially expressed between PXO99^A^ and PH inoculated leaf samples. This study highlighted the possible candidate genes that may play role in susceptibility in rice after *Xoo* infection.

## Additional file

Additional file 1: **Table S1.** Summary of RNA-Seq paired-end data produced by Illumina sequencing. **Table S2.** List of 1143 DEGs expressed at different time points in JG30 genotype after infection of PXO99^A^ and PH strains. The significant DEGs were retrieved by Log2FC ≥1 (up-regulated genes) or ≤ − 1 (down-regulated genes) as a threshold level. The gene ID, FPKM based expression pattern, GO terms, and chromosomal position are mentioned in the table. **Table S3.** A detailed list of TFs expressed differentially in JG30 genotype at different time points after PXO99^A^ and PH inoculation. **Table S4.** List of significant enriched different Kinase responsive genes differentially expressed at different time points in JG30 genotype after PXO99^A^ and PH strains. **Table S5.** A detailed list of differentially expressed peroxidase responsive genes in JG30 genotype after PXO99^A^ and PH inoculations at different time points. **Table S6.** Significant KEGG pathways (*P*-value ≤0.05) involved in JG30 genotype after PXO99^A^ and PH infection. **Table S7.** MapMan analysis of DEGs involved in biotic stress pathway in JG30 genotype. The gene ID, bin code, gene description, and average Log2FC values are presented in the below-mentioned table. **Table S8.** MapMan analysis of DEGs involved in different metabolic pathways in JG30 genotype. The gene ID, bin code, gene description, and average Log2FC values are presented in the below-mentioned table. **Table S9.** Summary of GO terms enriched in JG30 genotype after PXO99^A^ and PH infection. **Table S10.** A list of significantly enriched GO terms (P-value ≤0.05) having DEGs at different time points after infection of PXO99^A^ and PH strains. **Table S11.** A list of eight genes primers used for RNA-Seq data validation. (XLSX 359 kb)

## Data Availability

All the raw data of sequencing reads have been deposited in NCBI SRA database under the accession No. SAMN11081413.
